# Travel Medicine Curricula across Canadian Pharmacy Programs and Alignment with Scope of Practice

**DOI:** 10.3390/pharmacy8020102

**Published:** 2020-06-15

**Authors:** Heidi V.J. Fernandes, Brittany Cook, Sherilyn K.D. Houle

**Affiliations:** 1 School of Pharmacy, University of Waterloo, Waterloo, ON N2L 3G1, Canada; heidi.fernandes@uwaterloo.ca; 2 Health Sciences North, Sudbury, ON P3E 5J1, Canada; bcook@hsnsudbury.ca

**Keywords:** travel medicine, pharmacists, pharmacy education, curriculum, vaccination

## Abstract

Limited research exists on pharmacy students’ training in travel medicine, and how this aligns with scope of practice. This research aimed to detail travel medicine education across pharmacy programs in Canada and map this against the scope of practice for pharmacists in each university’s jurisdiction. A survey based on the International Society of Travel Medicine’s Body of Knowledge was developed and distributed to all Canadian undergraduate pharmacy schools to identify topic areas taught, teaching modalities utilized, and knowledge assessment performed. Educational data was collected and analyzed descriptively, and compared to pharmacists’ scope of practice in the province in which each university is located. Training provided to students varied significantly across universities and topic areas, with topics amenable to self-care (e.g., traveller’s diarrhea and insect bite prevention) or also encountered outside of the travel context (e.g., sexually transmitted infections) taught more regularly than travel-specific topics (e.g., dengue and altitude illness). No apparent relationship was observed between a program’s curriculum and their provincial scope of practice. For example, training in vaccine-preventable diseases did not necessarily align with scope related to vaccine administration. Alignment of education to current and future scope will best equip new practitioners to provide care to travelling patients.

## 1. Introduction

The scope of practice for pharmacists in Canada varies by province/territory; however, most jurisdictions have implemented expansions in scope that facilitate the provision of travel medicine care by pharmacists [1]. For example, in Alberta, pharmacists have the option to attain Additional Prescribing Authorization, which allows them to independently prescribe any vaccine as well as medications that are not narcotics or controlled drugs [2], provided that they feel competent in the therapeutic area they are prescribing within. In Manitoba, pharmacists with the Certificate in Travel Health^TM^ (CTH^®^) from the International Society of Travel Medicine (ISTM) and at least 1000 hours of experience can apply for Extended Practice Pharmacist status, which allows them to prescribe travel-related vaccines or medications [3]. For Alberta and Manitoba, typical travel medications that can be prescribed by pharmacists would include, but are not limited to, antibiotics for travellers’ diarrhea such as azithromycin, malaria chemoprophylaxis such as atovaquone/proguanil, and agents to prevent altitude illness such as acetazolamide [2,3]. In Ontario and British Columbia, pharmacists are able to administer travel vaccines but lack the authority to prescribe unless obtained through a medical directive [4,5]. In New Brunswick, all pharmacists can prescribe for “preventable diseases” including vaccines for cholera, hepatitis, measles, mumps, and rubella, and drugs for malaria and travellers’ diarrhea, while those with CTH^®^ can also prescribe vaccines against rabies, typhoid, Japanese encephalitis, and yellow fever [6]. Pharmacists in any province wishing to administer yellow fever vaccinations need to comply with regulations outlined by the Public Health Agency of Canada and have their pharmacy registered as a designated Yellow Fever Vaccination Centre [7]. As a result of these scope expansions, an increasing number of pharmacists are providing pre-travel consultations and/or administering travel-related vaccinations nationwide. Similar scope expansions have been enacted in other countries, including the United States (e.g., travel vaccine administration in 86.3% of jurisdictions, and prescribing medications in 52.9% of jurisdictions, usually under a collaborative practice agreement) [8], Switzerland (e.g., administration of certain travel vaccines in 13 of 26 cantons) [9], and the United Kingdom (e.g., supplementary and independent prescribing) [10]. Furthermore, in the United Kingdom, the 2017 change in status of Maloff Protect^®^ (atovaquone/proguanil hydrochloride) for adult use from prescription to over-the-counter status has created additional opportunities for pharmacists there to provide care to travellers [11].

Travel medicine poses unique challenges for health care professionals as a result of its complexity and need for highly individualized care, taking patient-, destination-, and itinerary-related factors into consideration. Complicating this further, we hypothesize that few faculties of medicine, nursing, or pharmacy educate students in travel medicine sufficiently to be able to practice in that area upon graduation, or be able to recognize signs and symptoms of travel-related health issues in general practice. Indeed, reports have been published of suboptimal documentation of travel history among patients presenting to emergency departments with symptoms that may be suggestive of tropical diseases [12,13,14]. Lack of travel medicine training in medical school has been reported as a contributing factor impacting the quality of travel-related care provided by general practitioners [15]. Similarly, Canadian pharmacists have reported insufficient knowledge and skills in travel medicine as a leading barrier limiting their involvement in the field [16,17,18]. These barriers are exacerbated by the fact that expertise in travel medicine must typically be self-acquired by clinicians through learning activities such as continuing education courses and practical experience in providing travel consultations. To address this, calls to action have been made for greater availability of training opportunities as part of undergraduate health professional education [19], such as the elective special study module offered to medical students at the National University of Ireland Galway [20]. 

To our knowledge, only one study has assessed travel medicine training within health professional programs, specifically focussed on schools of pharmacy in California [21]. This study found varying levels of coverage of topics across core and elective courses, ranging from 85.7% of programs providing instruction on travellers’ diarrhea to 28.6% providing instruction on post-travel assessment. Differences in scope of practice for pharmacists across Canada, coupled with variations in knowledge and skill obtained through self-study and continuing professional education, has the potential to result in variation in the quantity and quality of care provided by Canadian pharmacists. This study aims to identify the breadth and depth of instruction in travel medicine topics currently offered to Canadian pharmacy students, and map this against the scope of practice for pharmacists in each university’s respective province.

## 2. Materials and Methods 

### 2.1. Study Design and Population

A cross-sectional survey was conducted to capture the various areas of travel medicine that are covered in pharmacy curricula across the ten pharmacy schools in Canada [22].

### 2.2. Questionnaire Design

The questionnaire, available in Supplementary Table S1, was based on the competencies and content areas of the ISTM’s Body of Knowledge [23]. This list comprises the topics tested on the Certificate of Travel Health^TM^ examination—the internationally recognized designation of travel medicine expertise.

The ISTM’s Body of Knowledge is a comprehensive list of tropical diseases and other travel-related topics; however, as a multidisciplinary document, some topics may not be within the scope of practice of pharmacists, or may be less relevant to most pharmacists’ practices. We therefore excluded or modified the following sections/subsections to ensure appropriateness and brevity:

- As determining fitness to travel and psychological status, post-travel assessment, and medical care abroad are out of scope for pharmacists, these sections were omitted.
- For organization and brevity, the “types of vaccines” list was subdivided into routine, recommended, and travel vaccine categories. Routine vaccines were defined as those included in the immunization programs of most provinces and territories in Canada [24]. Recommended vaccines were defined as those generally recommended to the broad population but often not funded by the Canadian universal health care system. Travel vaccines were defined as those specifically indicated for travel outside of Canada according to the recommendations of the Committee to Advise on Tropical Medicine and Travel [25].
- Vaccine-related education was assumed to also include basic information on the disease being prevented by that vaccine.
- Within “special populations” and each of the travel-related disease categories, less common subtopics were combined into a single “other” category to keep the focus of the questionnaire on items most likely to be encountered by pharmacists in general practice.

The questionnaire was comprised of 61 items. Question types were largely categorical, with free text utilized to capture the teaching time associated with each topic area and to explain additional teaching activities not captured by our survey. For each item, respondents were asked whether:

1. The material is taught within the program (yes/no);
2. What course(s) the material is covered in;
3. Whether the course(s) is/are required or elective;
4. The mode(s) of teaching (options included in class/didactic, self-study/online, simulation labs, other);
5. Whether the students learning is evaluated in a summative assessment (yes/no);
6. Estimated total hours of teaching time across the entire topic section.

Prior to distribution, the questionnaire was reviewed for clarity by two pharmacists with experience in academia and travel medicine to ensure understandability and ease of use.

### 2.3. Data Collection and Ethics

One individual from each of the ten pharmacy programs in Canada was identified as the initial contact by reviewing the faculty listings on each institution’s website. Staff members with a focus (teaching or research) on travel medicine were contacted first at their institutional email address. This initial contact provided information on the study as well as a copy of the data collection form (as a fillable form in Microsoft Word^®^). If no staff members listed fit this description, the same information and data collection form were sent by email to a member of the program’s curriculum committee or a staff member whose online profile identified that they were involved in some way with the undergraduate curriculum. In the absence of any of this information or non-response from the individuals initially contacted, the program dean/director was emailed for guidance. One document was to be completed per program, recognizing that collaboration across multiple instructors may be required to collate the information requested. To increase response rates, multiple follow-up emails were sent, and alternate individuals were contacted as required as described above. Initial email invitations were sent in January 2019, with the last response received in September 2019.

This research complies with the Tri-Council Policy Statement: Ethical Conduct for Research Involving Humans [26], which states that “In some cases, research may involve interaction with individuals who are not themselves the focus of the research in order to obtain information” [22]. Although individuals were contacted to conduct this study, the data that was the focus of this study was of the institution rather than the individual. Although these individuals were contacted to conduct the study, they are not classified as human research participants. As such, ethics approval and participant consent was not required by our institution [27]; however, participation by those contacted was voluntary.

### 2.4. Data Analysis

Given the small number of pharmacy schools in Canada, all data was analyzed descriptively to determine the current landscape across the country regarding topics taught, by which method(s), how assessment was performed, and teaching time. This information was considered at the level of each university as well as across the country by comparing overall findings across all programs. Topics taught by each program were then compared to the scope of practice for the province within which each institution was located. Scope of practice for each province was determined by consulting the resources published by the Canadian Pharmacists Association [1], published literature on Canadian pharmacists’ scope of practice related to vaccination [28], and provincial regulatory body websites as required. In the assessment of alignment between teaching areas and scope of practice related to prescribing, only malaria, travellers’ diarrhea and altitude sickness were included due to the frequency at which they are seen in travel medicine practice and the frequent need for prescription products for their prevention and/or treatment [29]. For ease of analysis, the administration of injections was collated into the categories of routine, non-travel recommended, and travel vaccines, with the outcome being the percent of vaccines within each category taught in the program (recognizing as a limitation that some provinces/territories’ scope may include only some vaccines within each category and not all). To conceal the identity of each institution for publication, a university number was randomly assigned to each completed questionnaire by the individual performing data analysis. When a range was provided for hours of instruction, the midpoint was reported and used for any subsequent calculations.

## 3. Results

The survey had a completion rate of 80% (n = 8). The results are summarized in the Supplementary file, with the full responses provided in Supplementary Table S2.

Apart from the data obtained from the questionnaire, additional relevant feedback and program descriptions were provided. For example, University 4 was the only pharmacy program that offered an elective course dedicated to travel medicine. This course captured educational topics beyond those included in the questionnaire, particularly related to mental health related to travel (described as “traveling with comorbid psychiatric conditions, and the increased incidence of psychiatric decompensation abroad especially in younger travellers and expatriates”). Apart from didactic learning, University 2 also incorporates problem-based learning for their students and University 3 uses case discussions and small group learning activities. Lastly, the results obtained from University 7 were for their Bachelor’s degree program; however, they were in the midst of transitioning to an entry-to-practice PharmD degree and recognized that some topics, teaching and assessment methods, and teaching time may change within the new program.

### 3.1. Epidemiology

Almost all respondents (n = 7) teach basic concepts of epidemiology (e.g., morbidity, mortality) to their pharmacy learners. The majority of the respondents (n = 6) teach geographic specificity/global distribution of diseases and potential health hazards.

### 3.2. Immunology/Vaccinology

All respondents reported teaching on routine and recommended vaccines, including the use of summative assessments of student knowledge within required courses. However, the extent to which programs cover vaccines starts to vary when vaccines had overlap between immunizations that could be considered both routine and for travel. For example, while hepatitis A and B vaccination were taught by all eight institutions, poliomyelitis was taught by five, and rabies taught by four. Two programs (universities 2 and 7) indicated not providing any education on travel vaccines other than hepatitis.

### 3.3. Pre-Travel Assessment/Consultation

The two universities with limited instruction on travel vaccines also reported that they did not provide instruction on performing a pre-travel assessment/consultation, with the exception of relevant medical history (e.g., previous vaccinations, allergies, chronic illness, mental health history and concurrent medications), which was taught by University 2. However, they noted that this was part of their injection skills training and may not necessarily be focussed on medical history considerations specifically related to travel. 

Variation in coverage of special populations was observed across universities as follows: Pregnant travellers (n = 6), travellers with chronic diseases (n = 6), child travellers (n = 4), immunocompromised individuals (n = 4), travellers visiting friends and relatives (n = 3), immigrants (n = 2 programs). Other special populations (e.g., athletes, business travellers, elderly travellers, expatriates/long-term travellers) were taught by three programs—University 4’s elective course, in a required course for University 8, and in both a required and elective course for University 6.

Two universities provide instruction on travel health related to the Hajj pilgrimage; however, other special itineraries were only taught in elective courses offered by universities 4 and 6.

### 3.4. Diseases Contracted During Travel

#### 3.4.1. Diseases Associated with Vectors

Personal protective measures against insect bites are taught by all responding programs, across both required and elective courses. Malaria was the most widely taught vector-borne disease (n = 6), followed by Zika virus (n = 5), Lyme disease (n = 3), and West Nile Virus (n = 2). Three responding programs teach on at least one of the vector-borne diseases categorized as “Other” (e.g., chikungunya, dengue, Japanese encephalitis).

#### 3.4.2. Diseases Associated with Person-to-Person Contact

All responding programs cover sexually transmitted infections (STIs) and four teach students on tuberculosis—all within required courses. 

#### 3.4.3. Diseases Associated with Ingestion of Food and Water

All responding programs teach precautions to use to protect against diseases associated with ingestion of food and water in required courses, while University 4 also teaches it in an elective course. Travellers’ diarrhea is also taught by all responding programs. Cholera is taught by five programs, and is required learning by four of these programs. All but one program that teaches on cholera also teach typhoid and paratyphoid fever. “Other” topics within this section (amebiasis, brucellosis, cryptosporidiosis, cyclosporiasis, giardiasis, and norovirus) are taught by half of the responding programs.

#### 3.4.4. Diseases Associated with Bites and Stings

Rabies is taught in half of the responding programs—universities 1, 3, 4 and 6—as described above in Section 3.2. “Other” topics included envenomation and herpes B virus, which had at least one topic in this category taught by two programs—one within an elective course only, and one within both an elective and required course.

#### 3.4.5. Diseases Associated with Water/Environmental Contact

Each of these conditions was combined into a single category, with at least one topic covered within elective courses by University 4, and within required and elective courses by University 1.

### 3.5. Other Clinical Conditions Associated with Travel

Motion sickness, sun-induced damage/conditions, and thrombosis/embolism were the most taught conditions within this section, with seven institutions offering it mostly in required courses. A full breakdown of the exact offerings and modalities is available in Supplementary Table S2. Other conditions in this category include altitude sickness (n = 5), jet lag (n = 4), barotrauma (n = 3), and frostbite/hypothermia (n = 3). Interestingly, only one program teaches on respiratory distress/failure (associated with humidity, pollution, etc.) and this is through the elective travel medicine course offered by University 4.

### 3.6. Administrative and General Travel Medicine Issues

Accessing health information for travellers was taught by 75% of the responding programs (universities 1, 2, 3, 4, 6 and 8) within required courses (n = 4), elective courses (n = 1), or a combination of both (n = 1). International health regulations and national/regional recommendations were taught by 4 programs each. 

### 3.7. Teaching Modalities, Assessment, and Teaching Time

As indicated in Supplementary Table S2, most topics were delivered in class through didactic teaching, with the second most common teaching mode being online self-study. The majority of items were evaluated using summative assessment, with the exception of Universities 2 and 3 which provided some of the items for information only, or utilized formative assessment only. In total, 21 of the 61 items were delivered by at least one institution using simulations labs, predominantly by universities 4 and 6, and typically in combination with didactic teaching. The total teaching time dedicated to travel medicine ranged from 6.75 to 46.5 hours (mean 24.4, SD 13.5).

### 3.8. Instruction of Topics in Relation to Provincial Scope of Practice

No association appeared between a university’s travel medicine curriculum and the scope of practice for pharmacists in their respective province. For example, the province within which University 7 is located allows pharmacists to inject any vaccine and prescribe within a collaborative practice/agreement, yet no travel vaccines are covered in their program apart from hepatitis A and B. Conversely, University 4 teaches all the vaccinations listed in our survey, but pharmacists in that province cannot independently prescribe those that are Schedule I (i.e., requiring a prescription), which comprises many travel vaccines [30]. The full comparison of practice scope to travel medicine curricula can be found in Supplementary Table S3. 

## 4. Discussion

A high degree of variability exists in the teaching of travel medicine topics across pharmacy schools in Canada. It may therefore be hypothesized that variation in exposure to these topics during pharmacy school contributes to variation in practice following graduation. Overall, an association was noted between instruction on a particular travel medicine topic and its potential overlap with another area of pharmacy practice. For example, motion sickness is a common minor ailment [31] that many community pharmacists encounter in daily practice both within and outside the context of international travel. Similarly, STIs can be acquired both domestically and abroad and, therefore, is a core component of many infectious disease curricula. These are both examples of topics included in the ISTM’s Body of Knowledge that were taught by all participating programs (n = 8) that can traverse beyond the realm of travel medicine. 

No clear relationship was observed between a pharmacy’s program travel medicine curriculum and its respective province’s scope of pharmacy practice. For example, University 4 was found to be the only program with its own travel medicine elective for pharmacy students and subsequently taught all the items listed in our survey. However, its province does not allow certain activities relating to travel medicine such as the independent ability to prescribe drugs for malaria chemoprophylaxis. Similarly, programs located in provinces where pharmacists can administer any drug or vaccine did not teach 100% of the immunizations included in our survey. It was also outside the scope of this study to determine whether level of instruction is correlated with likelihood to practice in travel medicine following graduation. Finally, while the ISTM recognizes the value of practical learning by suggesting the accumulation of 2–3 years of practice experience before writing their Certificate in Travel Health^TM^ (CTH^®^) exam [32], teaching using simulation was uncommon, with only 21% of the travel medicine items included in our survey taught by simulation by at least one institution.

Pharmacy programs in Canada are accredited by The Canadian Council for Accreditation of Pharmacy Programs (CCAPP). While teaching of specific therapeutic areas is not mandated by this body, Criterion 4.1 requires that “The curriculum has content of sufficient depth, scope, timeliness, quality, sequence and emphasis to provide the foundation for the full scope of contemporary pharmacy practice responsibilities as well as emerging roles” [33]. While not all pharmacists may opt to provide advanced travel services, one can argue that basic competency in pre-travel assessment (particularly recognition of situations requiring referral of the patient to a professional with travel medicine expertise) and care related to travel medicine should be considered both an existing and emerging role. Pharmacy programs are therefore encouraged to reflect upon their current curriculum related to travel medicine to ensure new graduates are sufficiently prepared to embrace these roles and to ensure that accreditation expectations can be satisfied now and in the future.

While the questionnaire was purposely sent to individuals with sufficient knowledge about the program’s curriculum, we cannot rule out reporting error, misinterpretation, or incomplete responses by the representative completing the survey. While this study provides a strong curricular map representing eight pharmacy programs in nine different provinces, it does not represent all Canadian pharmacy schools due to non-response from two programs. A further limitation is the lack of differentiation within the information we were trying to gather from the universities. Some topics are relevant both domestically and with travel (e.g., epidemiology, clinical history-taking, STIs, tuberculosis), and we did not distinguish whether these topics were taught within the context of travel. This limitation could explain large discrepancies in teaching hours observed among institutions for the same topic. For example, University 1 reported spending 0.75 hours teaching epidemiology whereas University 2 reported 10 hours of instruction time on this topic. We also did not differentiate teaching hours for a given topic taught across both required and elective courses. Instead, the total teaching time was asked for each participating school and the individual breakdown between required or elective courses were not obtained. Therefore, the total teaching hours may not be an accurate representation of instruction received by all students versus by only some. Additionally, as teaching hours were self-reported by each institution, reporting of time spent by students on topics taught through self-directing learning may be inaccurate from the students’ perspective, or inconsistent across institutions. Finally, as a cross-sectional study, these results reflect training provided to new pharmacy graduates and do not capture changes to pharmacy curricula over time (in other words, teaching provided to more experienced pharmacists).

To our knowledge, this is the first comprehensive curricular review of travel medicine education across pharmacy professional programs. Previous publications have been limited to one jurisdiction and were less comprehensive in topics included [21,34]. However, similar to our work, gaps in training in pharmacy professional programs were noted by these studies, and are consistent with studies of pharmacists that report that the primary modality used to acquire travel medicine knowledge is continuing professional education programming [16,18]. The results of our study can help pharmacy programs in Canada and internationally to reflect on their travel medicine teaching to ensure pharmacists are equipped to address common scenarios that may be encountered in practice. It also provides support for the delivery of continuing professional development programs by advocacy and professional interest organizations, as many travel-related topics are not taught routinely across most undergraduate pharmacy programs. Additional research on perceived readiness to practice specifically in travel-related areas among new graduates can identify additional areas for improvement within pharmacy curricula and for continuing professional development programs. While readiness to administer injections in general and to perform other tasks such as adapting prescriptions among new graduates in Ontario, Canada, has been documented [35], similar work has not been done specifically looking at travel medicine. Finally, these results support pharmacists’ self-report of feeling inadequately knowledgeable to provide travel-related care [16,17,18]. As patients value travel consultations performed by pharmacists [29,36,37] and these services become more common, undergraduate and post-graduate training programs must recognize and respond to these educational needs to equip pharmacists to practice to their full scope.

## 5. Conclusions

This is the first study mapping travel medicine education in pharmacy curricula in Canada. While not all universities in Canada responded, our results showed that there was a high degree of variability among the pharmacy programs with travel medicine concepts that comprise the ISTM’s Body of Knowledge. Additionally, there was no apparent relationship between a pharmacy program’s travel medicine education and their respective province’s scope of pharmacy practice. Undergraduate and post-graduate training opportunities in travel medicine would benefit from expansion to mirror expansions in pharmacist scope related to travel medicine practice.

## Supplementary Materials

The following are available online at https://www.mdpi.com/2226-4787/8/2/102/s1, Table S1: Travel Medicine Curriculum Survey, Table S2: Travel Medicine Curriculum Survey Results, Table S3: Comparison of Scopes of Practice and Travel Medicine Curricula.
