# Supplementary material for: Travel Medicine Curricula across Canadian Pharmacy Programs and Alignment with Scope of Practice
Source: Pharmacy (Basel). 2020 Jun 15;8(2):102. doi: 10.3390/pharmacy8020102 (PMC7355656; doi:10.3390/pharmacy8020102)
Supplement: Supplementary file 1 [file pharmacy-08-00102-s001.pdf]

Supplementary Table S1. Travel Medicine Curriculum Survey

Epidemiology.

		If "Yes" to "Taught in Program"			
**Topic**	**Taught in Program**	**Course Name**	**Type of Course**	**Teaching Mode(s) SELECT ALL THAT APPLY**	**Is student learning evaluated in a summative assessment?**
Basic concepts (e.g., morbidity, mortality, incidence, prevalence)	☐ Yes<br>☐ No		☐ Required/Core<br>☐ Elective	☐ In Class/Didactic<br>☐ Self-Study/Online<br>☐ Simulation Labs<br>☐ Other:	☐ Yes<br>☐ No (for information only, or formative assessment)
Geographic specificity/global distribution of disease and potential health hazards	☐ Yes<br>☐ No		☐ Required/Core<br>☐ Elective	☐ In Class/Didactic<br>☐ Self-Study/Online<br>☐ Simulation Labs<br>☐ Other:	☐ Yes<br>☐ No (for information only, or formative assessment)
Estimated total hours of teaching time on **epidemiology** topics above: ___________					

Immunology/Vaccinology.

		If "Yes" to "Taught in Program"			
**Topic**	**Taught in Program**	**Course Name**	**Type of Course**	**Teaching Mode(s) SELECT ALL THAT APPLY**	**Is student learning evaluated in a summative assessment?**
Basic concepts (e.g., live vs. inactivated vaccine, measurement of immune response)	☐ Yes<br>☐ No		☐ Required/Core<br>☐ Elective	☐ In Class/Didactic<br>☐ Self-Study/Online<br>☐ Simulation Labs<br>☐ Other:	☐ Yes<br>☐ No (for information only, or formative assessment)
Handling, storage, and disposal of vaccines and related supplies	☐ Yes<br>☐ No		☐ Required/Core<br>☐ Elective	☐ In Class/Didactic<br>☐ Self-Study/Online<br>☐ Simulation Labs<br>☐ Other:	☐ Yes<br>☐ No (for information only, or formative assessment)
**Routine vaccination (disease information, indications/contraindications, routes of administration, dosing regimens, duration of protection, immunogenicity, efficacy, potential adverse reactions and their management)**					
Bacille Calmette–Guérin	☐ Yes<br>☐ No		☐ Required/Core<br>☐ Elective	☐ In Class/Didactic<br>☐ Self-Study/Online<br>☐ Simulation Labs<br>☐ Other:	☐ Yes<br>☐ No (for information only, or formative assessment)
Diptheria, tetanus, pertussis	☐ Yes<br>☐ No		☐ Required/Core<br>☐ Elective	☐ In Class/Didactic<br>☐ Self-Study/Online<br>☐ Simulation Labs<br>☐ Other:	☐ Yes<br>☐ No (for information only, or formative assessment)
Haemophilus influenzae type B	☐ Yes<br>☐ No		☐ Required/Core<br>☐ Elective	☐ In Class/Didactic<br>☐ Self-Study/Online<br>☐ Simulation Labs<br>☐ Other:	☐ Yes<br>☐ No (for information only, or formative assessment)
Influenza	☐ Yes<br>☐ No		☐ Required/Core<br>☐ Elective	☐ In Class/Didactic<br>☐ Self-Study/Online<br>☐ Simulation Labs<br>☐ Other:	☐ Yes<br>☐ No (for information only, or formative assessment)
Measles, mumps, rubella	☐ Yes<br>☐ No		☐ Required/Core<br>☐ Elective	☐ In Class/Didactic<br>☐ Self-Study/Online<br>☐ Simulation Labs<br>☐ Other:	☐ Yes<br>☐ No (for information only, or formative assessment)

Meningococcal	☐ Yes<br>☐ No	☐ Required/Core<br>☐ Elective	☐ In Class/Didactic<br>☐ Self-Study/Online<br>☐ Simulation Labs<br>☐ Other:	☐ Yes<br>☐ No (for information only, or formative assessment)
Poliomyelitis	☐ Yes<br>☐ No	☐ Required/Core<br>☐ Elective	☐ In Class/Didactic<br>☐ Self-Study/Online<br>☐ Simulation Labs<br>☐ Other:	☐ Yes<br>☐ No (for information only, or formative assessment)
Varicella	☐ Yes<br>☐ No	☐ Required/Core<br>☐ Elective	☐ In Class/Didactic<br>☐ Self-Study/Online<br>☐ Simulation Labs<br>☐ Other:	☐ Yes<br>☐ No (for information only, or formative assessment)
Estimated total hours of teaching time on **routine vaccinations** above: ___________				
**Non-travel recommended vaccinations (indications/contraindications, routes of administration, dosing regimens, duration of protection, immunogenicity, efficacy, potential adverse reactions and their management):**				
Human papillomavirus	☐ Yes<br>☐ No	☐ Required/Core<br>☐ Elective	☐ In Class/Didactic<br>☐ Self-Study/Online<br>☐ Simulation Labs<br>☐ Other:	☐ Yes<br>☐ No (for information only, or formative assessment)
Pneumococcal	☐ Yes<br>☐ No	☐ Required/Core<br>☐ Elective	☐ In Class/Didactic<br>☐ Self-Study/Online<br>☐ Simulation Labs<br>☐ Other:	☐ Yes<br>☐ No (for information only, or formative assessment)
Tetanus	☐ Yes<br>☐ No	☐ Required/Core<br>☐ Elective	☐ In Class/Didactic<br>☐ Self-Study/Online<br>☐ Simulation Labs<br>☐ Other:	☐ Yes<br>☐ No (for information only, or formative assessment)
Zoster	☐ Yes<br>☐ No	☐ Required/Core<br>☐ Elective	☐ In Class/Didactic<br>☐ Self-Study/Online<br>☐ Simulation Labs<br>☐ Other:	☐ Yes<br>☐ No (for information only, or formative assessment)
Estimated total hours of teaching time on **non-travel recommended vaccinations** above: ___________				

**Travel vaccinations (indications/contraindications, routes of administration, dosing regimens, duration of protection, immunogenicity, efficacy, potential adverse reactions and their management):**				
Cholera	☐ Yes<br>☐ No	☐ Required/Core<br>☐ Elective	☐ In Class/Didactic<br>☐ Self-Study/Online<br>☐ Simulation Labs<br>☐ Other:	☐ Yes<br>☐ No (for information only, or formative assessment)
Japanese encephalitis	☐ Yes<br>☐ No	☐ Required/Core<br>☐ Elective	☐ In Class/Didactic<br>☐ Self-Study/Online<br>☐ Simulation Labs<br>☐ Other:	☐ Yes<br>☐ No (for information only, or formative assessment)
Hepatitis A, hepatitis B	☐ Yes<br>☐ No	☐ Required/Core<br>☐ Elective	☐ In Class/Didactic<br>☐ Self-Study/Online<br>☐ Simulation Labs<br>☐ Other:	☐ Yes<br>☐ No (for information only, or formative assessment)
Rabies	☐ Yes<br>☐ No	☐ Required/Core<br>☐ Elective	☐ In Class/Didactic<br>☐ Self-Study/Online<br>☐ Simulation Labs<br>☐ Other:	☐ Yes<br>☐ No (for information only, or formative assessment)
Tick-borne encephalitis	☐ Yes<br>☐ No	☐ Required/Core<br>☐ Elective	☐ In Class/Didactic<br>☐ Self-Study/Online<br>☐ Simulation Labs<br>☐ Other:	☐ Yes<br>☐ No (for information only, or formative assessment)
Typhoid	☐ Yes<br>☐ No	☐ Required/Core<br>☐ Elective	☐ In Class/Didactic<br>☐ Self-Study/Online<br>☐ Simulation Labs<br>☐ Other:	☐ Yes<br>☐ No (for information only, or formative assessment)
Yellow fever	☐ Yes<br>☐ No	☐ Required/Core<br>☐ Elective	☐ In Class/Didactic<br>☐ Self-Study/Online<br>☐ Simulation Labs<br>☐ Other:	☐ Yes<br>☐ No (for information only, or formative assessment)
Estimated total hours of teaching time on **travel vaccinations** above: ___________				

Pre-Travel Assessment/Consultation.

Topic	Taught in Program	If "Yes" to "Taught in Program"			
		**Course Name**	**Type of Course**	**Teaching Mode(s) SELECT ALL THAT APPLY**	**Is student learning evaluated in a summative assessment?**
**Patient evaluation**					
Evaluation of travel itineraries/risk assessment (e.g., pre-existing activities, travel to rural vs. urban areas)	☐ Yes<br>☐ No		☐ Required/Core<br>☐ Elective	☐ In Class/Didactic<br>☐ Self-Study/Online<br>☐ Simulation Labs<br>☐ Other:	☐ Yes<br>☐ No (for information only, or formative assessment)
Relevant medical history (e.g., previous vaccinations, allergies, chronic illness, mental health history, and concurrent medications)	☐ Yes<br>☐ No		☐ Required/Core<br>☐ Elective	☐ In Class/Didactic<br>☐ Self-Study/Online<br>☐ Simulation Labs<br>☐ Other:	☐ Yes<br>☐ No (for information only, or formative assessment)
Estimated total hours of teaching time on **patient evaluation** topics above: ___________					
**Special populations and the unique management issues pertaining to the following populations:**					
Immigrants	☐ Yes<br>☐ No		☐ Required/Core<br>☐ Elective	☐ In Class/Didactic<br>☐ Self-Study/Online<br>☐ Simulation Labs<br>☐ Other:	☐ Yes<br>☐ No (for information only, or formative assessment)
Infants and children	☐ Yes<br>☐ No		☐ Required/Core<br>☐ Elective	☐ In Class/Didactic<br>☐ Self-Study/Online<br>☐ Simulation Labs<br>☐ Other:	☐ Yes<br>☐ No (for information only, or formative assessment)
Pregnant travellers and nursing mothers	☐ Yes<br>☐ No		☐ Required/Core<br>☐ Elective	☐ In Class/Didactic<br>☐ Self-Study/Online<br>☐ Simulation Labs<br>☐ Other:	☐ Yes<br>☐ No (for information only, or formative assessment)
Travellers with chronic diseases (e.g., diabetes, chronic obstructive pulmonary disease, cardiovascular disease, mental health issues)	☐ Yes<br>☐ No		☐ Required/Core<br>☐ Elective	☐ In Class/Didactic<br>☐ Self-Study/Online<br>☐ Simulation Labs<br>☐ Other:	☐ Yes<br>☐ No (for information only, or formative assessment)
Travellers who are immunocompromised, including AIDS and HIV	☐ Yes<br>☐ No		☐ Required/Core<br>☐ Elective	☐ In Class/Didactic<br>☐ Self-Study/Online<br>☐ Simulation Labs	☐ Yes<br>☐ No (for information only, or formative assessment)

			☐ Other:	
VFRs (those visiting friends and relatives in their countries of origin)	☐ Yes<br>☐ No	☐ Required/Core<br>☐ Elective	☐ In Class/Didactic<br>☐ Self-Study/Online<br>☐ Simulation Labs<br>☐ Other:	☐ Yes<br>☐ No (for information only, or formative assessment)
**Others:** Athletes, business travellers, elderly travellers, expatriates/long-term travellers, travel for the purpose of international adoption, missionaries/health workers, travellers with disabilities, travel to hostile environments	☐ Yes<br>☐ No	☐ Required/Core<br>☐ Elective	☐ In Class/Didactic<br>☐ Self-Study/Online<br>☐ Simulation Labs<br>☐ Other:	☐ Yes<br>☐ No (for information only, or formative assessment)
Estimated total hours of teaching time on **special population** topics above: ___________				
**Special itineraries and the unique management issues associated with the following activities/itineraries:**				
Mass gatherings (e.g., the Hajj)	☐ Yes<br>☐ No	☐ Required/Core<br>☐ Elective	☐ In Class/Didactic<br>☐ Self-Study/Online<br>☐ Simulation Labs<br>☐ Other:	☐ Yes<br>☐ No (for information only, or formative assessment)
**Others**: Armed conflict zones, cruise travel/sailing, diving, extended stay travel, extreme/wilderness travel, last-minute travel, travel to receive medical care, natural disaster areas, sex tourism, areas experiencing disease outbreaks	☐ Yes<br>☐ No	☐ Required/Core<br>☐ Elective	☐ In Class/Didactic<br>☐ Self-Study/Online<br>☐ Simulation Labs<br>☐ Other:	☐ Yes<br>☐ No (for information only, or formative assessment)
Estimated total hours of teaching time on **special itineraries** topics above: ___________				

Travel-Related Diseases.

		If "Yes" to "Taught in Program"			
**Topic**	**Taught in Program**	**Course Name**	**Type of Course**	**Teaching Mode(s) SELECT ALL THAT APPLY**	**Is student learning evaluated in a summative assessment?**
**Diseases associated with vectors (including risk, prevention, signs/symptoms, complications, treatment):**					
Lyme	☐ Yes<br>☐ No		☐ Required/Core<br>☐ Elective	☐ In Class/Didactic<br>☐ Self-Study/Online<br>☐ Simulation Labs<br>☐ Other:	☐ Yes<br>☐ No (for information only, or formative assessment)
Malaria	☐ Yes<br>☐ No		☐ Required/Core<br>☐ Elective	☐ In Class/Didactic<br>☐ Self-Study/Online<br>☐ Simulation Labs<br>☐ Other:	☐ Yes<br>☐ No (for information only, or formative assessment)
West Nile	☐ Yes<br>☐ No		☐ Required/Core<br>☐ Elective	☐ In Class/Didactic<br>☐ Self-Study/Online<br>☐ Simulation Labs<br>☐ Other:	☐ Yes<br>☐ No (for information only, or formative assessment)
Yellow Fever	☐ Yes<br>☐ No		☐ Required/Core<br>☐ Elective	☐ In Class/Didactic<br>☐ Self-Study/Online<br>☐ Simulation Labs<br>☐ Other:	☐ Yes<br>☐ No (for information only, or formative assessment)
Zika	☐ Yes<br>☐ No		☐ Required/Core<br>☐ Elective	☐ In Class/Didactic<br>☐ Self-Study/Online<br>☐ Simulation Labs<br>☐ Other:	☐ Yes<br>☐ No (for information only, or formative assessment)
**Others:** African tick-bite fever, chikungunya, dengue, Japanese encephalitis, tick-borne encephalitis, filariasis, hemorrhagic fevers, leishmaniasis, plague, typhus, Rift Valley fever, trypanosomiasis	☐ Yes<br>☐ No		☐ Required/Core<br>☐ Elective	☐ In Class/Didactic<br>☐ Self-Study/Online<br>☐ Simulation Labs<br>☐ Other:	☐ Yes<br>☐ No (for information only, or formative assessment)
Personal protective measures against bites	☐ Yes<br>☐ No		☐ Required/Core<br>☐ Elective	☐ In Class/Didactic<br>☐ Self-Study/Online<br>☐ Simulation Labs	☐ Yes<br>☐ No (for information only, or formative assessment)

			☐ Other:	
Estimated total hours of teaching time on **diseases associated with vectors** topics above: ___________				
**Diseases associated with person-to-person contact (including risk, prevention, signs/symptoms, complications, treatment)**				
Sexually transmitted diseases	☐ Yes<br>☐ No	☐ Required/Core<br>☐ Elective	☐ In Class/Didactic<br>☐ Self-Study/Online<br>☐ Simulation Labs<br>☐ Other:	☐ Yes<br>☐ No (for information only, or formative assessment)
Tuberculosis	☐ Yes<br>☐ No	☐ Required/Core<br>☐ Elective	☐ In Class/Didactic<br>☐ Self-Study/Online<br>☐ Simulation Labs<br>☐ Other:	☐ Yes<br>☐ No (for information only, or formative assessment)
Estimated total hours of teaching time on **diseases associated with person-to-person contact** above: ___________				
**Diseases associated with ingestion of food and water (including risk, prevention, signs/symptoms, complications, treatment)**				
Cholera	☐ Yes<br>☐ No	☐ Required/Core<br>☐ Elective	☐ In Class/Didactic<br>☐ Self-Study/Online<br>☐ Simulation Labs<br>☐ Other:	☐ Yes<br>☐ No (for information only, or formative assessment)
Travellers' diarrhea	☐ Yes<br>☐ No	☐ Required/Core<br>☐ Elective	☐ In Class/Didactic<br>☐ Self-Study/Online<br>☐ Simulation Labs<br>☐ Other:	☐ Yes<br>☐ No (for information only, or formative assessment)
Typhoid and paratyphoid fever	☐ Yes<br>☐ No	☐ Required/Core<br>☐ Elective	☐ In Class/Didactic<br>☐ Self-Study/Online<br>☐ Simulation Labs<br>☐ Other:	☐ Yes<br>☐ No (for information only, or formative assessment)
**Others:** Amebiasis, brucellosis, cryptosporidiosis, cyclosporiasis, giardiasis, norovirus	☐ Yes<br>☐ No	☐ Required/Core<br>☐ Elective	☐ In Class/Didactic<br>☐ Self-Study/Online<br>☐ Simulation Labs<br>☐ Other:	☐ Yes<br>☐ No (for information only, or formative assessment)
Food and water precautions	☐ Yes<br>☐ No	☐ Required/Core<br>☐ Elective	☐ In Class/Didactic<br>☐ Self-Study/Online<br>☐ Simulation Labs<br>☐ Other:	☐ Yes<br>☐ No (for information only, or formative assessment)
Estimated total hours of teaching time on **disease associated with ingestion of food and water** topics above: ___________				

Diseases associated with bites and stings (including risk, prevention, signs/symptoms, complications, treatment):				
Rabies	☐ Yes<br>☐ No	☐ Required/Core<br>☐ Elective	☐ In Class/Didactic<br>☐ Self-Study/Online<br>☐ Simulation Labs<br>☐ Other:	☐ Yes<br>☐ No (for information only, or formative assessment)
**Others**: Envenomation (e.g., jelly fish, sea urchin, scorpion, snake, spiders), herpes B virus, cutaneous larva migrans, legionella, leptospirosis, schistosomiasis	☐ Yes<br>☐ No	☐ Required/Core<br>☐ Elective	☐ In Class/Didactic<br>☐ Self-Study/Online<br>☐ Simulation Labs<br>☐ Other:	☐ Yes<br>☐ No (for information only, or formative assessment)
Estimated total hours of teaching time on **diseases associated with bites and stings** topics above: ___________				

Other Clinical Conditions Associated with Travel.

		If "Yes" to "Taught in Program"			
**Topic**	**Taught in Program**	**Course Name**	**Type of Course**	**Teaching Mode(s) SELECT ALL THAT APPLY**	**Is student learning evaluated in a summative assessment?**
Barotrauma	☐ Yes<br>☐ No		☐ Required/Core<br>☐ Elective	☐ In Class/Didactic<br>☐ Self-Study/Online<br>☐ Simulation Labs<br>☐ Other:	☐ Yes<br>☐ No (for information only, or formative assessment)
Jet lag	☐ Yes<br>☐ No		☐ Required/Core<br>☐ Elective	☐ In Class/Didactic<br>☐ Self-Study/Online<br>☐ Simulation Labs<br>☐ Other:	☐ Yes<br>☐ No (for information only, or formative assessment)
Motion sickness	☐ Yes<br>☐ No		☐ Required/Core<br>☐ Elective	☐ In Class/Didactic<br>☐ Self-Study/Online<br>☐ Simulation Labs<br>☐ Other:	☐ Yes<br>☐ No (for information only, or formative assessment)
Thrombosis/embolism	☐ Yes<br>☐ No		☐ Required/Core<br>☐ Elective	☐ In Class/Didactic<br>☐ Self-Study/Online<br>☐ Simulation Labs<br>☐ Other:	☐ Yes<br>☐ No (for information only, or formative assessment)
Altitude sickness	☐ Yes<br>☐ No		☐ Required/Core<br>☐ Elective	☐ In Class/Didactic<br>☐ Self-Study/Online<br>☐ Simulation Labs	☐ Yes<br>☐ No (for information only, or formative assessment)

				☐ Other:	
Frostbite and hypothermia	☐ Yes<br>☐ No		☐ Required/Core<br>☐ Elective	☐ In Class/Didactic<br>☐ Self-Study/Online<br>☐ Simulation Labs<br>☐ Other:	☐ Yes<br>☐ No (for information only, or formative assessment)
Respiratory distress/failure (associated with humidity, pollution, etc.)	☐ Yes<br>☐ No		☐ Required/Core<br>☐ Elective	☐ In Class/Didactic<br>☐ Self-Study/Online<br>☐ Simulation Labs<br>☐ Other:	☐ Yes<br>☐ No (for information only, or formative assessment)
Sunburn, heat exhaustion, and sun stroke	☐ Yes<br>☐ No		☐ Required/Core<br>☐ Elective	☐ In Class/Didactic<br>☐ Self-Study/Online<br>☐ Simulation Labs<br>☐ Other:	☐ Yes<br>☐ No (for information only, or formative assessment)
Estimated total hours of teaching time on **other clinical conditions associated with travel** topics above: ___________					

Travel Medicine Information/Resources.

				If "Yes" to "Taught in Program"	
**Topic**	**Taught in Program**	**Course Name**	**Type of Course**	**Teaching Mode(s) SELECT ALL THAT APPLY**	**Is student learning evaluated in a summative assessment?**
Accessing health information for travellers including commercial and proprietary sources	☐ Yes<br>☐ No		☐ Required/Core<br>☐ Elective	☐ In Class/Didactic<br>☐ Self-Study/Online<br>☐ Simulation Labs<br>☐ Other:	☐ Yes<br>☐ No (for information only, or formative assessment)
International health regulations	☐ Yes<br>☐ No		☐ Required/Core<br>☐ Elective	☐ In Class/Didactic<br>☐ Self-Study/Online<br>☐ Simulation Labs<br>☐ Other:	☐ Yes<br>☐ No (for information only, or formative assessment)
National/regional recommendations, including national/regional differences	☐ Yes<br>☐ No		☐ Required/Core<br>☐ Elective	☐ In Class/Didactic<br>☐ Self-Study/Online<br>☐ Simulation Labs<br>☐ Other:	☐ Yes<br>☐ No (for information only, or formative assessment)
Estimated total hours of teaching time on **travel medicine information/resources** topics above: ___________					

Supplementary Table S2. Travel Medicine Curriculum Survey Results

Epidemiology.

	University 1	University 2	University 3	University 4	University 5	University 6	University 7	University 8
**Basic concepts**	✓	✓	✓	✓	✓	✓	×	✓
Course	R	R	R	E	R	R		R
Teaching mode(s)	S	D, PBL	D	D	S	D, S		D
Assessment	SUM	SUM	FORM	SUM	SUM	SUM		SUM
**Global distribution of disease**	✓	×	✓	✓	✓	✓	×	✓
Course	R		R	E	R	R		R
Teaching mode(s)	D, S		D	D	S	D, S		D
Assessment	SUM		FORM	SUM	SUM	SUM		SUM
**Teaching time (hours)**	0.75	10†	1	1	1	0.5	0	0.5

Immunology/Vaccinology.

	University 1	University 2	University 3	University 4	University 5	University 6	University 7	University 8
**Basic concepts and principles**	✓	✓	✓	✓	✓	✓	✓	✓
Course	R	R	R	R, E	R	R	R	R
Teaching mode(s)	S	D	D, S	D	S	D, S	D	S
Assessment	SUM	SUM	SUM	SUM	SUM	SUM	SUM	SUM
**Handling, storage, disposal of vaccines/supplies**	✓	✓	✓	✓	✓	✓	×	×
Course	R	R	R	R	R	R		
Teaching mode(s)	S, L	D, S, L	D, L	S	D, S	D, S		
Assessment	SUM	SUM	SUM	SUM	SUM	SUM		

Routine Vaccines.

	University 1	University 2	University 3	University 4	University 5	University 6	University 7	University 8
**Bacille Calmette–Guérin**	✗	✗	✗	✓	✗	✓	✓	✗
Course				E		R	R	
Teaching mode(s)				D		D, S	D	
Assessment				SUM		SUM	SUM	
**Diphtheria, tetanus, pertussis**	✓	✓	✓	✓	✓	✓	✓	✓
Course	R	R	R	R, E	R	R	R	R
Teaching mode(s)	S	S	D, C, G	D	S	D, S	D	D
Assessment	SUM	FORM	SUM	SUM	SUM	SUM	SUM	SUM
**Haemophilus influenzae type B**	✓	✓	✓	✓	✓	✓	✓	✓
Course	R	R	R	R, E	R	R	R	R
Teaching mode(s)	S	D, PBL	D, C, G	D	D, S	D, S	D	D
Assessment	SUM	SUM	SUM	SUM	SUM	SUM	SUM	SUM
**Influenza**	✓	✓	✓	✓	✓	✓	✓	✓
Course	R	R	R	R, E	R	R	R	R
Teaching mode(s)	D, S	D, PBL	D, C, G	D, L	D, S	D, S	D	D
Assessment	SUM	SUM	SUM	SUM	SUM	SUM	SUM	SUM
**Measles, mumps, rubella**	✓	✓	✓	✓	✓	✓	✓	✓
Course	R	R	R	R, E	R	R	R	R
Teaching mode(s)	S	S	D, C, G	D	S	D, S	D	D
Assessment	SUM	FORM	SUM	MISSING	SUM	SUM	SUM	SUM
**Meningococcal**	✓	✓	✓	✓	✓	✓	✓	✓
Course	R, E	R	R	R, E	R	R	R	R
Teaching mode(s)	S	D, PBL	D, C, G	D	D, S	D, S	D	D
Assessment	SUM, FORM	SUM	SUM	SUM	SUM	SUM	SUM	SUM
**Poliomyelitis**	✓	✗	✓	✓	✗	✓	✗	✓
Course	R		R	R, E		R		R
Teaching mode(s)	D, S		D, C, G	D		D, S		D
Assessment	SUM		SUM	SUM		SUM		SUM
**Varicella**	✓	✓	✓	✓	✓	✓	✓	✓
Course	R	R	R	R, E	R	R	R	R
Teaching mode(s)	S	S	D, C, G	D, L	D, S	D, S	D	D
Assessment	SUM	FORM	SUM	SUM	SUM	SUM	SUM	SUM
**Teaching time (hours)**	4	6 *	2.5	4	1	2.5	3	1

Recommended Vaccines.

	University 1	University 2	University 3	University 4	University 5	University 6	University 7	University 8
**Human papillomavirus**	✓	✓	✓	✓	✓	✓	✓	✓
Course	R	R	R	R, E	R	R	R	R
Teaching mode(s)	D, S	D, PBL	D, C, G	D, L	D, S	D, S	D	D
Assessment	SUM	SUM	SUM	SUM	SUM	SUM	SUM	SUM
**Pneumococcal**	✓	✓	✓	✓	✓	✓	✓	✓
Course	R	R	R	R, E	R	R	R	R
Teaching mode(s)	D, S	D, PBL	D, C, G	D, L	D, S	D, S	D	D
Assessment	SUM	SUM	SUM	SUM	SUM	SUM	SUM	SUM
**Tetanus**	✓	✓	✓	✓	✓	✓	✓	✓
Course	R	R	R	E	R	R	R	R
Teaching mode(s)	S	S	D, C, G	D	S	D, S	MISSING	D
Assessment	SUM	FORM	SUM	SUM	SUM	SUM	MISSING	SUM
**Herpes zoster**	✓	✓	✓	✓	✓	✓	✓	✓
Course	R	R	R	R, E	R	R	E	R
Teaching mode(s)	S	D, PBL	D, C, G	D, L	D, S	D, S	D	D
Assessment	SUM	SUM	SUM	SUM	SUM	SUM	SUM	SUM
**Teaching time (hours)**	1.5	3*	2.5	1	0.5	0.5	2	1

Travel Vaccines.

	University 1	University 2	University 3	University 4	University 5	University 6	University 7	University 8
**Cholera**	✓	×	✓	✓	✓	✓	×	✓
Course	R, E		R	R, E	R	R		R
Teaching mode(s)	D, S		D, S	D	D, S	D, S		D
Assessment	SUM, FORM		FORM	SUM	SUM	SUM		SUM

**Hepatitis (A/B)**	✓	✓	✓	✓	✓	✓	✓	✓
Course	R, E	R	R	R, E	R	R	R	R
Teaching mode(s)	D, S	D	D, S, C, G	D	D, S	D, S	D	D
Assessment	SUM, FORM	SUM	SUM	SUM	SUM	SUM	SUM	SUM
**Japanese encephalitis**	✓	×	✓	✓	×	✓	×	✓
Course	R, E		R	E		R		R
Teaching mode(s)	D, S		D, S	D		D, S		D
Assessment	SUM, FORM		FORM	SUM		SUM		SUM
**Rabies**	✓	×	✓	✓	×	✓	×	×
Course	R, E		R	E		R		
Teaching mode(s)	S		D	D		D, S		
Assessment	SUM, FORM		FORM	SUM		SUM		
**Tick-borne encephalitis**	×	×	×	✓	×	×	×	✓
Course				E				R
Teaching mode(s)				D				D
Assessment				SUM		SUM		SUM
**Typhoid**	✓	×	✓	✓	✓	✓	×	✓
Course	R, E		R	R, E	R	R		R
Teaching mode(s)	D, S		D, S	D	D, S	D, S		D
Assessment	SUM, FORM		FORM	SUM	SUM	MISSING		SUM
**Yellow fever**	✓	×	✓	✓	✓	✓	MISSING	✓
Course	R, E		R	R, E	R	R	MISSING	R
Teaching mode(s)	S		D, S	D	D, S	D, S	MISSING	D
Assessment	SUM		FORM	SUM	SUM	SUM	MISSING	SUM
**Teaching time (hours)**	1	1.5	2	7	0.5	0.75	0.5	1

Pre-Travel Consultation.

	University 1	University 2	University 3	University 4	University 5	University 6	University 7	University 8
**Patient evaluation**	✓	×	✓	✓	✓	✓	×	✓
Course	R		R	E	R	R		R
Teaching mode(s)	D, S		D, C	D	D	D, S, L		D
Assessment	SUM		SUM	SUM	SUM	SUM		SUM
**Medical history**	✓	✓	✓	✓	✓	✓	×	✓
Course	R	R	R	E	R	R		R
Teaching mode(s)	D, S	D	D, C	D	D	D, S, L		D
Assessment	SUM	SUM, FORM	SUM	SUM	SUM	SUM		SUM
**Teaching time (hours)**	1	0.5-1	0.5	2	0.25	2.5	0	MISSING

Special Populations.

	University 1	University 2	University 3	University 4	University 5	University 6	University 7	University 8
**Immigrants**	×	×	×	✓	✓	×	×	×
Course				E	R			
Teaching mode(s)				D	S			
Assessment				SUM	SUM			
**Children**	×	×	×	✓	✓	✓	×	✓
Course				R, E	R	R		R
Teaching mode(s)				D	S	D, S		D, L
Assessment				SUM	SUM	SUM		SUM
**Pregnancy**	✓	×	✓	✓	✓	✓	×	✓
Course	R		R	R, E	R	R		R
Teaching mode(s)	S		D, C	D	S	D, S		D
Assessment	SUM		SUM	SUM	SUM	SUM		SUM

**Chronic disease**	✓	×	✓	✓	✓	✓	×	✓
Course	R		R	E	R	R		R
Teaching mode(s)	S		D, C	D	S	D, S		D
Assessment	SUM		SUM	SUM	SUM	SUM		SUM
**Immunocompromised**	✓	×	×	✓	✓	✓	×	×
Course	R			E	R	R		
Teaching mode(s)	S			D	S	D, S		
Assessment	SUM			SUM	SUM	SUM		
**Visiting friends and relatives**	✓	×	✓	✓	×	×	×	×
Course	R		R	E				
Teaching mode(s)	D		D	D				
Assessment	SUM		FORM	SUM				
**Other**	×	×	×	✓	×	✓	×	✓
Course				E		R, E		R
Teaching mode(s)				D		D, S		D
Assessment				SUM		SUM		SUM
**Teaching time (hours)**	1	0	0.5	6	1	0.5	0	1

Special Itineraries.

	**University 1**	**University 2**	**University 3**	**University 4**	**University 5**	**University 6**	**University 7**	**University 8**
**Hajj**	✓	×	×	✓	×	×	×	×
Course	R			E				
Teaching mode(s)	D			D				
Assessment	SUM			SUM				
**Other**	×	×	×	✓	×	✓	×	×
Course				E		E		
Teaching mode(s)				D		D, S		
Assessment				SUM		SUM		
**Teaching time (hours)**	0.1	0	0	2	0	2	0	0

Diseases associated with vectors (including risk, prevention, signs/symptoms, complications, treatment).

	University 1	University 2	University 3	University 4	University 5	University 6	University 7	University 8
**Lyme**	×	✓	×	✓	✓	×	×	×
Course		R		E	R			
Teaching mode(s)		D		D	D			
Assessment		SUM		SUM	SUM			
**Malaria**	✓	×	✓	✓	✓	✓	×	✓
Course	R, E		R	R, E	R	R		R
Teaching mode(s)	D		D, C	D, L	D	D, S		D
Assessment	SUM		SUM	SUM	SUM	SUM		SUM
**West Nile**	×	×	×	✓	×	×	×	✓
Course				E				R
Teaching mode(s)				D				D
Assessment				SUM				SUM
**Yellow fever**	✓	×	✓	✓	✓	×	×	×
Course	R, E		R	E	R			
Teaching mode(s)	D, S		D	D	D			
Assessment	SUM		FORM	SUM	SUM			
**Zika**	✓	×	✓	✓	✓	✓	×	×
Course	R, E		R	E	R	R		
Teaching mode(s)	D		D, C	D	D	L		
Assessment	SUM		SUM	SUM	SUM	SUM		
**Other**	✓	×	✓	✓	×	×	×	×
Course	R		R	E				
Teaching mode(s)	D		D	D				
Assessment	SUM		FORM	MISSING				
**Personal protective measures**	✓	✓	✓	✓	MISSING	✓	✓	✓
Course	R, E	MISSING	R	R, E	MISSING	R	R	R
Teaching mode(s)	D, S, L	MISSING	D, C	D, L	MISSING	D, S, L	D	D
Assessment	SUM	MISSING	SUM	SUM	MISSING	SUM	SUM	SUM
**Teaching time (hours)**	0.75	1	1.5	8	0.5	2	0.5	1

Diseases associated with person-to-person contact (including risk, prevention, signs/symptoms, complications, treatment).

	University 1	University 2	University 3	University 4	University 5	University 6	University 7	University 8
**Sexually transmitted infections**	✓	✓	✓	✓	✓	✓	✓	✓
Course	R, E	R	R	R, E	R	R, E	R	R
Teaching mode(s)	D, S	D, PBL	D, G	D	D	D, S, L	D	D
Assessment	SUM	SUM	SUM	SUM	SUM	SUM	SUM	SUM
**Tuberculosis**	×	×	✓	✓	×	✓	MISSING	✓
Course			R	R, E		R	MISSING	R
Teaching mode(s)			D	D		D, S	MISSING	D
Assessment			SUM	SUM		SUM	MISSING	SUM
**Teaching time (hours)**	2	7.5*	5	1	0.5	10	6	6

Diseases associated with ingestion of food and water (including risk, prevention, signs/symptoms, complications, treatment).

	University 1	University 2	University 3	University 4	University 5	University 6	University 7	University 8
**Cholera**	✓	×	✓	✓	✓	✓	×	×
Course	R		R	E	R	R		
Teaching mode(s)	D		D	D	D	D, S		
Assessment	SUM		FORM	SUM	SUM	SUM		
**Travellers' diarrhea**	✓	✓	✓	✓	✓	✓	✓	✓
Course	R, E	R	R	R, E	R	R	R	R
Teaching mode(s)	D, S	D, PBL	D, C	D, L	D	D, S, L	D	D
Assessment	SUM	SUM	MISSING	SUM	SUM	SUM	SUM	SUM
**Typhoid**	✓	×	✓	✓	✓	×	×	×
Course	R		R	R, E	R			
Teaching mode(s)	D, S		D	D	D			
Assessment	SUM		FORM	MISSING	SUM			
**Other**	×	×	✓	✓	✓	✓	×	×
Course			R	E	R	R		
Teaching mode(s)			D	D	D	D, S		
Assessment			SUM	SUM	SUM	SUM		
**Food and water precautions**	✓	✓	✓	✓	✓	✓	✓	✓

Course	R	R	R	R, E	R	R	R	MISSING
Teaching mode(s)	S	D	D, C	L	D	D, S, L	D	MISSING
Assessment	SUM	SUM	SUM	SUM	SUM	SUM	SUM	MISSING
**Teaching time (hours)**	1	1-2*	1.5	3	0.5	3.5	MISSING	1

Diseases associated with bites and stings (including risk, prevention, signs/symptoms, complications, treatment).

	University 1	University 2	University 3	University 4	University 5	University 6	University 7	University 8
**Rabies**	✓	×	✓	✓	×	✓	×	×
Course	R		R	E		R		
Teaching mode(s)	S		D	D		L		
Assessment	SUM		SUM	SUM		SUM		
**Other**	✓	×	×	✓	×	×	×	×
Course	R, E			E				
Teaching mode(s)	D			D				
Assessment	SUM			SUM				
**Teaching time (hours)**	0.25	0	0.25	3	0	0.5	0	0

Other Conditions Associated with Travel.

	University 1	University 2	University 3	University 4	University 5	University 6	University 7	University 8
**Barotrauma**	×	×	×	✓	✓	×	✓	×
Course				E	R		R	
Teaching mode(s)				D	D		D	
Assessment				SUM	SUM		SUM	
**Jet lag**	✓	×	×	✓	×	✓	✓	×
Course	R			E		R	R	
Teaching mode(s)	D			D, L		L	D	
Assessment	SUM			SUM		SUM	SUM	
**Motion sickness**	✓	✓	✓	✓	✓	×	✓	✓
Course	E	R	R	R, E	R		R	R
Teaching mode(s)	S, L	D	D	D, L	D		D	D
Assessment	SUM	SUM	SUM	SUM	SUM		SUM	SUM

**Embolism/Thrombosis**	✓	✓	✓	✓	×	✓	✓	✓
Course	R	R	R	E		R	R	R
Teaching mode(s)	D	D, PBL	D	D		L	D	D
Assessment	SUM	SUM	SUM	SUM		SUM	SUM	SUM
**Altitude sickness**	✓	×	✓	✓	×	✓	×	✓
Course	R, E		R	R, E		R		R
Teaching mode(s)	D		D	D, L		D, S, L		D
Assessment	SUM		FORM	SUM		SUM		SUM
**Frostbite and hypothermia**	✓	×	×	✓	×	×	✓	×
Course	R			E			R	
Teaching mode(s)	D			D			D	
Assessment	SUM			SUM			SUM	
**Respiratory distress/failure**	×	×	×	✓	×	×	MISSING	×
Course				E			MISSING	
Teaching mode(s)				D			MISSING	
Assessment				SUM			MISSING	
**Sunburn, heat exhaustion, sun stroke**	✓	×	✓	✓	✓	✓	✓	✓
Course	R		R	E	R	R	MISSING	R
Teaching mode(s)	D, S		D	D, L	D, L	D, S	MISSING	D
Assessment	SUM		MISSING	SUM	SUM	SUM	MISSING	SUM
**Teaching time (hours)**	3	8.5*	2	3	1	3	3.5	6

Travel Medicine Information/Resources.

	University 1	University 2	University 3	University 4	University 5	University 6	University 7	University 8
**Accessing health information for travellers**	✓	✓	✓	✓	×	✓	×	✓
Course	R, E	R	R	E		R		R
Teaching mode(s)	D, S	D, S	D, G	D		D, S		D
Assessment	SUM	FORM	FORM	SUM		SUM		SUM
**International health regulations**	✓	×	×	✓	×	✓	×	✓
Course	R			E		E		R
Teaching mode(s)	D			D		D, S		D
Assessment	SUM			SUM		SUM		SUM

**National/regional recommendations**	✓	✓	✓	✓	×	×	×	×
Course	R, E	R	R	E				
Teaching mode(s)	D, S	D, S	D, G	D				
Assessment	SUM	SUM	FORM	SUM				
**Teaching time (hours)**	0.25	4-6	0.5	1	0	2.5	0	0.2

Total Teaching Time across All Topics.

	University 1	University 2	University 3	University 4	University 5	University 6	University 7	University 8
**Teaching Time (hours)**	16.6	43-46.5*	19.75	42	6.75	30.75	15.5	18.7

C = case discussions; D = didactic; E = elective; FORM = formative; G = small group activity; L = lab; PBL = problem-based learning; R = required; S = self-study; SUM = summative. † includes all therapeutic topics in curriculum; * excludes self-directed learning time.

Supplementary Table S3. Comparison of Scopes of Practice and Travel Medicine Curricula

Scope of Practice	University 1	University 2	University 3	University 4	University 5	University 6	University 7	University 8
**Prescribing**								
Prescribe independently for any Schedule I drug	No	No	Yes	No	No	No	No	No
Prescribe in a collaborative practice setting/agreement	No	Yes	Yes	No	No	No	Yes	No
Malaria taught	Yes	No	Yes	Yes	Yes	Yes	No	Yes
Travellers' diarrhea taught	Yes	Yes	Yes	Yes	Yes	Yes	Yes	Yes
Altitude sickness taught	Yes	No	Yes	Yes	No	Yes	No	Yes
**Immunizations**								
Inject any drug or vaccine	No	No	Yes	Pending	Yes	Pending	Yes	Pending
Inject vaccines only	Yes	Yes	Yes	Yes	Yes	Pending	Yes	Pending
Inject influenza vaccine	Yes	Yes	Yes	Yes	Yes	Pending	Yes	Pending
Percentage of routine vaccinations taught (includes influenza)	88% (n = 7)	75% (n = 6)	88% (n = 7)	100% (n = 8)	75% (n = 6)	100% (n = 8)	88% (n = 7)	88% (n = 7)
Percentage of non-travel recommended vaccinations taught	100% (n = 4)	100% (n = 4)	100% (n = 4)	100% (n = 4)	100% (n = 4)	100% (n = 4)	100% (n = 4)	100% (n = 4)
Percentage of travel vaccines taught	86% (n = 6)	13% (n = 1)	86% (n = 6)	100% (n = 7)	57% (n = 4)	86% (n = 6)	0% (n = 0)	86% (n = 6)
